# Aflatoxin B_1_ Degradation by a *Pseudomonas* Strain

**DOI:** 10.3390/toxins6103028

**Published:** 2014-10-23

**Authors:** Lancine Sangare, Yueju Zhao, Yawa Minnie Elodie Folly, Jinghua Chang, Jinhan Li, Jonathan Nimal Selvaraj, Fuguo Xing, Lu Zhou, Yan Wang, Yang Liu

**Affiliations:** 1 Institute of Agro-products Processing Science and Technology, Chinese Academy of Agricultural Sciences, Beijing 100193, China; E-Mails: lancin.sangar@gmail.com (L.S.); zhaoyueju@caas.cn (Y.Z.); yelodieminnie@yahoo.fr (Y.M.E.F.); cjh2975@sina.com (J.C.); lijinhan1990@163.com (J.L.); sjonnim@gmail.com (J.N.S.); xingfuguo@caas.cn (F.X.); zhoulu@caas.cn (L.Z.); awangyan@126.com (Y.W.); 2 Key Laboratory of Agro-products Processing, Ministry of Agriculture, Beijing 100193, China

**Keywords:** aflatoxin, degradation, culture supernatant, *Pseudomonas aeruginosa* N17-1

## Abstract

Aflatoxin B_1_ (AFB_1_), one of the most potent naturally occurring mutagens and carcinogens, causes significant threats to the food industry and animal production. In this study, 25 bacteria isolates were collected from grain kernels and soils displaying AFB_1_ reduction activity. Based on its degradation effectiveness, isolate N17-1 was selected for further characterization and identified as *Pseudomonas aeruginosa*. *P. aeruginosa* N17-1 could degrade AFB_1_, AFB_2_ and AFM_1_ by 82.8%, 46.8% and 31.9% after incubation in Nutrient Broth (NB) medium at 37 °C for 72 h, respectively. The culture supernatant of isolate N17-1 degraded AFB_1_ effectively, whereas the viable cells and intra cell extracts were far less effective. Factors influencing AFB_1_ degradation by the culture supernatant were investigated. Maximum degradation was observed at 55 °C. Ions Mn^2+^ and Cu^2+^ were activators for AFB_1_ degradation, however, ions Mg^2+^, Li^+^, Zn^2+^, Se^2+^, Fe^3+^ were strong inhibitors. Treatments with proteinase K and proteinase K plus SDS significantly reduced the degradation activity of the culture supernatant. No degradation products were observed based on preliminary LC-QTOF/MS analysis, indicating AFB_1_ was metabolized to degradation products with chemical properties different from that of AFB_1_. The results indicated that the degradation of AFB_1_ by *P. aeruginosa* N17-1 was enzymatic and could have a great potential in industrial applications. This is the first report indicating that the isolate of *P. aeruginosa* possesses the ability to degrade aflatoxin.

## 1. Introduction

Aflatoxins are a group of secondary metabolites mainly produced by the fungi *Aspergillus parasiticus*, *A. flavus*, *A. nomius*, *A. tamari* and *A. pseudotamarii* [1,2,3]. Aflatoxins, especially aflatoxin B_1_ (AFB_1_), are known for their carcinogenic, teratogenic, hepatotoxic and immunosuppressive effects on humans and animals [4,5]. Aflatoxins contamination in food and feed results in huge worldwide economic losses each year [6].

Several studies on physical and chemical strategies for the reduction of aflatoxins have been reported [7]. Nevertheless, none of these strategies completely fulfils the necessary efficacy, safety and cost requirements [8,9]. These disadvantages encouraged recent emphasis on the biological degradation of aflatoxins. Biological detoxification of AFB_1_ by fungal and bacterial isolates or their secondary metabolites has been reported, such as *Armillariella tabescens* [10,11], *Pleurotus ostreatus* [12], *Bacillus licheniformis* [13], *Bacillus subtilis* ANSB060 [14], *Mycobacterium fluoranthenivorans* sp. [15,16], *Myxococcus fulvus* [17], *Nocardia corynebacterioides* (formerly *Flavobacterium aurantiacum*) [18,19], *Rhodococcus erythropolis* [16,20,21], and *Stenotrophomonas maltophilia* [22]. AFB_1_ biodegradation by microorganisms and their metabolites, especially enzymes, is specific, effective and environmentally sound [17].

In an attempt to acquire new AFB_1_ degradation bacteria, we isolated microbes capable of degrading AFB_1_ from soils and contaminated kernels using coumarin medium. An efficient strain N17-1 of *Pseudomonas* was isolated and it displayed strong degradation activity on aflatoxin. The objectives of the present study were to (1) evaluate degradation efficiency of strain N17-1 on AFB_1_ and AFB_2_; (2) determine the degradation efficiency of N17-1 by culture supernatant, cell extracts and viable cells; (3) examine the factors affecting degradation efficiency of culture supernatant; and (4) make a preliminary analysis of degradation products.

## 2. Results

### 2.1. Screening for AFB_1_ Degradation Microbes

Twenty five bacterial isolates, obtained from 247 samples collected from different sources could reduce AFB_1_ concentration in NB after 3 days incubation at 37 °C with various degrees of effectiveness (Table S1). Three isolates showed more than 70% reduction in AFB_1_ in the medium. N17-1 is one of these isolates displaying higher level of AFB_1_ degradation ability by 82.8%.

### 2.2. Identification of Isolate N17-1

Isolate N17-1 showed the typical characteristics of *Pseudomonas aeruginosa*. The morphological, biochemical and physiological characteristics of isolate N17-1 were listed in Table S2. According to 16S rRNA gene sequence analysis, it was found that the closest relatives of strain N17-1 was *Pseudomonas* sp. KGS (99%). Based on the results of morphologic, physiological, biochemical characteristics and 16S rRNA gene sequence analysis, isolate N17-1 was finally identified as *Pseudomonas aeruginosa* N17-1. The partial 16S rRNA gene sequence of strain N17-1 was submitted to the database of GenBank, and the accession number is KJ188250. This N17-1 strain was deposited in China General Microbiological Culture Collection Center as CGMCC 8511.

### 2.3. AFB_1_ Degradation by Strain N17-1

**Figure 1:**
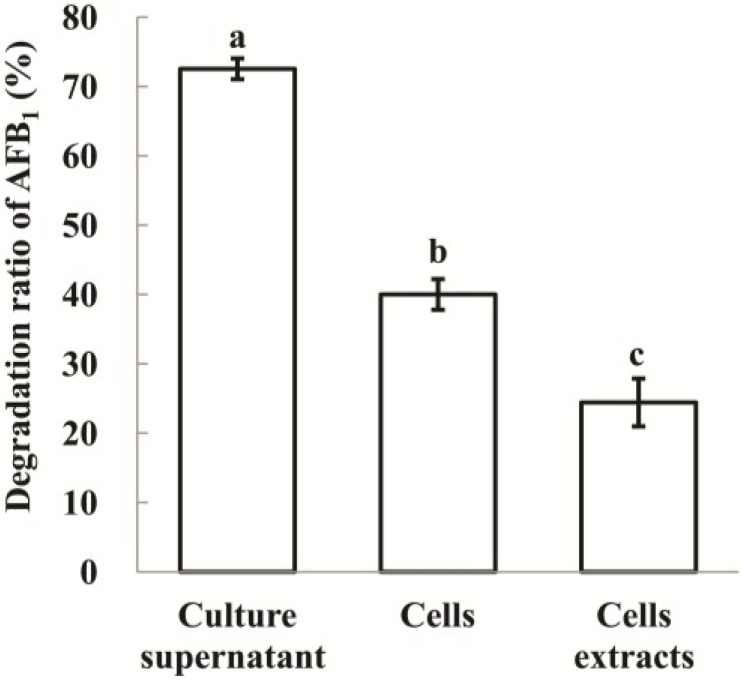
AFB_1_ degradation by culture supernatant, cell and cell extracts of *P. aeruginosa* N17-1 after 72 h incubation. The values are means of three replicates and their standard errors. Means with different letters are significantly different according to Duncan’s Multiple Range Test (*p* < 0.05).

Culture supernatant of strain N17-1 could degrade 72.5% AFB_1_ after 72 h incubation compared to 40.0% and 24.4% by viable cells and cell extracts, respectively. Culture supernatant was more effective (*p* < 0.05) than viable cells and cell extracts (Figure 1).

**Figure 2:**
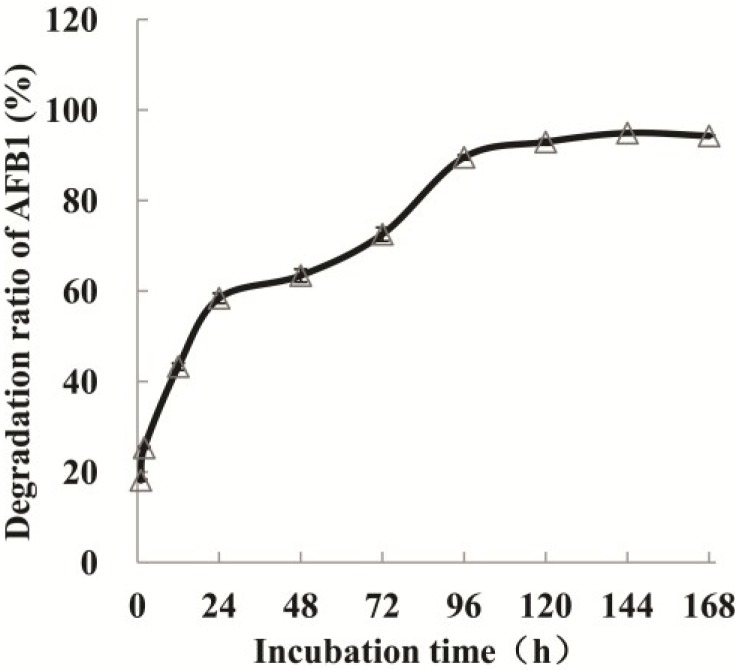
Dynamics of AFB_1_ degradation by *P. aeruginosa* N17-1 culture supernatant with time.

AFB_1_ degradation by the culture supernatant of strain N17-1 after different time intervals was investigated. AFB_1_ reduction ratio was 43.3% in the first 12 h, while 72.5% was degraded after 72 h. After 7 days, 94.3% AFB_1_ was degraded (Figure 2).

**Figure 3:**
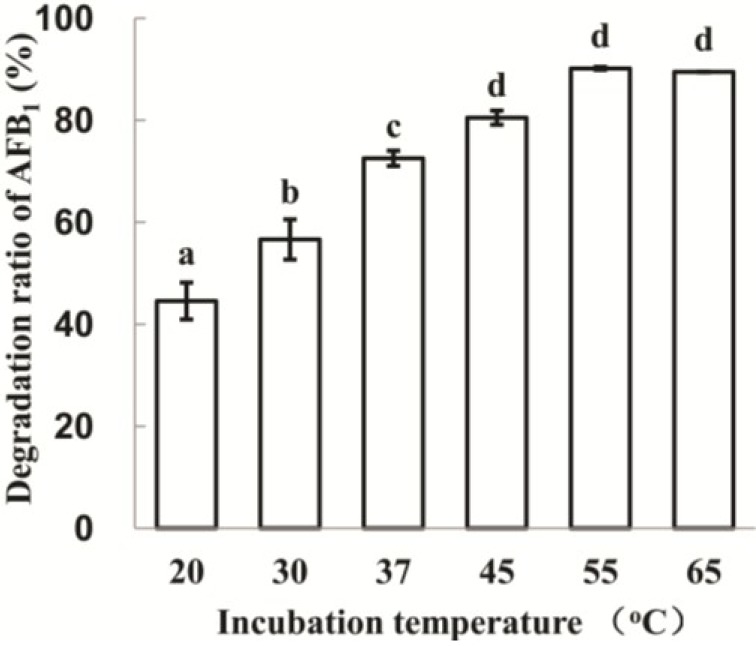
Effect of temperature on AFB_1_ degradation by culture supernatant of *P. aeruginosa* N17-1. To determine the effect of temperature, the mixtures were incubated at 20, 30, 37, 45, 55 and 65 °C, respectively for 72 h. The values are means of three replicates and their standard errors. Means with different letters are significantly different according to Duncan’s Multiple Range Test (*p* < 0.05).

The AFB_1_ degradation by strain N17-1 culture supernatant varied under different temperatures (Figure 3). The optimum degradation temperature was 55 °C (90.2%).

**Figure 4:**
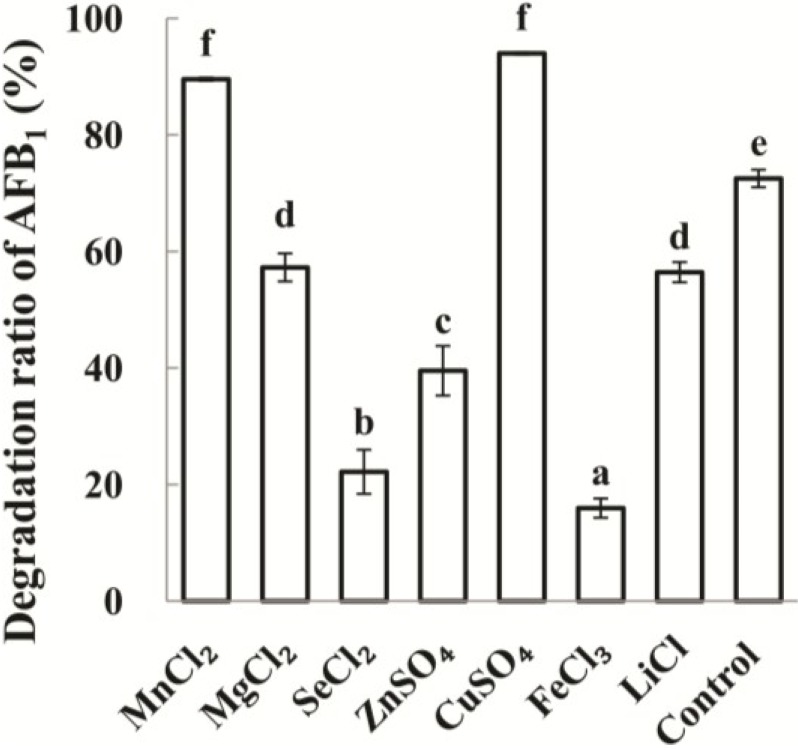
Effects of ions on AFB_1_ degradation by culture supernatant of *P. aeruginosa* N17-1. The values are means of three replicates and their standard errors. Means with different letters are significantly different according to Duncan’s Multiple Range Test (*p* < 0.05).

Metal ions could significantly affect AFB_1_ degradation (Figure 4). Mn^2+^ and Cu^2+^ stimulated AFB_1_ degradation at the concentration of 10 mM when compared with control (original concentration of AFB_1_ is 100 ppb). Their degradation rates were 89.6% and 94.0%, respectively. When compared with control, Mn^2+^ and Cu^2+^ increased AFB_1_ degradation level by 23.5% and 29.6%, respectively. However, Mg^2+^, Li^+^, Zn^2+^, Se^2+^ ions at 10 mM reduced the degradation ratio to 57.3%, 56.5%, 39.5% and 22.2%. Fe^3+^ ions inhibited the activity even more significantly, with only 16.0% of AFB_1_ degraded after 72 h. When AFB_1_ degradation ability of culture supernatant was tested with 10 mM Cu^2+^ at 55 °C for 72 h, no AFB_1_ could be observed.

It was found that the aflatoxin degradation ability of the culture supernatant increased by 46.3% after ultra filtration, which has a positive correlation with protein concentration (Table 1). When culture supernatant was treated with proteinase K, degradation AFB_1_ ability was decreased by 12.3%. When culture supernatant was treated with proteinase K plus SDS, degradation activity was significantly decreased by 34.0%. All these results implied that proteins or enzymes might be involved in the degradation by strain N17-1. Besides, when culture supernatant was treated by heat (boiling water bath for 10 min), degradation activity did not decrease, indicating proteins or enzymes involved in AFB_1_ degradation by strain N17-1 were heat-stable.

**Table 1:** Degradation of AFB_1_ by culture supernatant of strain N17-1 after 24 h incubation.

Supernatant conditions	Protein concentration (mg/mL)	Degradation (%)
**Culture supernatant**	0.24 ± 0.03	50.48 ± 2.40
**Super filtered culture supernatant ^a^**	1.22 ± 0.04	73.84 ± 1.65
**Boiled culture supernatant**	0.12 ± 0.02	52.24 ± 1.74

^a^ Culture supernatant was concentrated with super filter with cut-off molecular weight of 3 KD (Millipore).

### 2.4. Aflatoxin Degradation Ability of Strain N17-1

Besides AFB_1_, AFB_2_ and AFM_1_ degradation ability of strain N17-1 was also checked. N17-1 was able to degrade AFB_2_ and AFM_1_ after incubation in NB medium at 37 °C for 72 h, respectively. The degradation percentages of AFB_2_ and AFM_1_ were 46.8% and 31.9%, which are lower than that of AFB_1_. When AFB_2_ and AFM_1_ degradation ability of culture supernatant was tested with 10 mM Cu^2+^ at 55 °C for 72 h, no AFB_2_ and AFM_1_ could be observed.

### 2.5. Degradation Products Analysis

**Figure 5:**
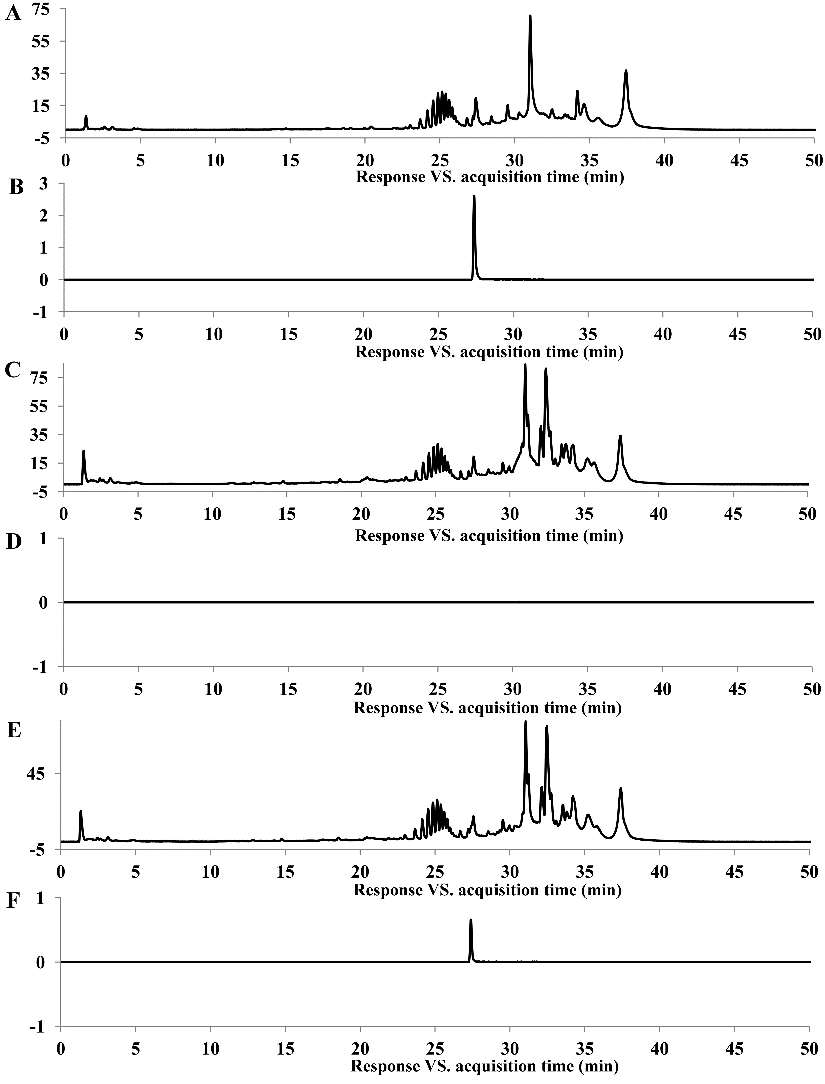
LC-QTOF/MS profile of ZON degradation. (**A**) Electrospray ionization (ESI)total ion chromatogram (TIC) scan of positive control (0.15 mL AFB_1_ (50 ppm) solution was added to 1.35 mL NB for final concentration of 5 ppm. After incubation in the dark at 37 °C for 72 h, the samples were extracted with chloroform); (**B**) ESI extracted ion chromatogram (EIC) scan of positive control; (**C**) ESI TIC scan of negative control (0.15 mL methanol instead of AFB_1_ solution was used as negative control); (**D**) ESI EIC scan of negative control; (**E**) ESI TIC scan of sample (0.15 mL AFB_1_ (50 ppm) solution was added to 1.35 mL culture supernatant of N17-1 for final concentration of 5 ppm. After incubation in the dark at 37 °C for 72 h, the samples were extracted with chloroform); (**F**) ESI EIC scan of sample.

To detect the main degradation products of AFB_1_, the concentration of AFB_1_ in culture supernatant of strain N17-1 was increased to 5 ppm. After 72 h incubation at 37 °C, degradation ratio of AFB_1_ was 67.0%. Degradation products were extracted by chloroform and further analyzed by LC-QTOF/MS. No degradation products could be observed, when compared with the negative and positive control by using the Agilent data analysis software and molecular feature extraction functions (MFE) for automatic database retrieval (Figure 5).

## 3. Discussion

In our study, a previous reported selection method with medium containing coumarin as the sole carbon source was used to select AFB_1_-degrading microbes from soils and cereal grains. A total of 25 isolates displaying various AFB_1_-degrading abilities were obtained, indicating that this method was selective and accurate. Taken together, three isolates could degrade AFB_1_ at a rate of more than 70% belonging to two genera, while ten isolates were obtained in Guan’s report [22] belonging to 8 genera, including *Stenotrophomonas* sp., *Brevundimonas* sp., *Bacillus* sp., *Klebsiella* sp., *Enterobacter* sp., *Brachybacterium* sp., *Rhodococcus* sp. and *Cellulosimicrobium* sp. Compared with Guan’s report, no genus displaying AFB_1_ reduction activity was shared in our research, which was probably caused by different sources of samples.

N17-1 was identified as *Pseudomonas aeruginosa*. It has been reported that isolates from this species could degrade *n*-Alkanes and Polycyclic Aromatic Hydrocarbons, benzene, toluene, xylene, acephate, methamidophos, decabromodiphenyl ether and edible oils [23,24,25,26,27]. However, this is the first report indicating that the isolate of this species possesses the ability to degrade aflatoxin.

Similar to *R. erythropolis* and *S. maltophilia* 35-3, degradation of AFB_1_ by the culture supernatant produced without pre-exposure to AFB_1_ was a constitutive activity of *P. aeruginosa* N17-1. Results implied that a protein (enzyme) or proteins (enzymes) might be involved in the degradation by *P. aeruginosa* N17-1. Various enzymes produced by *P. aeruginosa* are involved in the catabolic pathways of aromatic compounds via a cascade of reactions [23,24,25,26,27]. AFB_1_ is also a polyaromatic compound and could be degraded in a similar manner. The increase in detoxification by strain N17-1 with time indicated that a protein (enzyme) or proteins (enzymes) for AFB_1_ reduction could be stable at 37 °C for a week. The stability would bring practical benefits and be useful for application purpose.

Similar to *F. aurantiacum* and *S. maltophilia* 35-3, degradation of AFB_1_ by the culture supernatant of N17-1 could be stimulated by Cu^2+^ and inhibited by Zn^2+^ [22,28]. Instead of stimulation, Mg^2+^ ions showed inhibiting effect on AFB_1_ degradation.

Based on the results of super filtration, the AFB_1_ degradation ability of N17-1 culture supernatant is positively correlated with protein concentration in some content. It is indicated that proteins are involved in AFB_1_ degradation. Correspondently, proteinase K or proteinase K with SDS could significantly decrease the degradation ability of culture supernatant. Similar trends were observed in AFB_1_ degradation by culture supernatant of *Rhodococcus erythropolis*, *M. fulvus* ANSM068 and *S. maltophilia* [17,20,22].

Different microbes could convert AFB_1_ to different products. In white-rot fungus *Phanerochaete sordid* YK-624, AFB_1_ is first oxidized to AFB_1_-8,9-epoxide by manganese peroxidase and then hydrolyzed to AFB_1_-8,9-dihydrodiol [29]. In *Mycobacterium smegmatis*, enzymes utilize the deazaflavin cofactor F_420_H_2_ to catalyze the reduction of the α,β-unsaturated ester moiety of aflatoxins, activating the molecules for spontaneous hydrolysis and detoxification. Several low abundance metabolites possibly generated by spontaneous hydrolysis could be observed by LCMS [30]. In *A. tabescens* (E-20), the aflatoxin-detoxifizyme could change the structure of AFB_1_ observed by UV-visible spectrum [10]. *M. fulvus* ANSM068 could convert AFB_1_ to a new product. Further LCMS and IR analysis indicated biotransformation of AFB_1_ may be caused by modification of the lactone ring [17]. In the current study, we could not find any breakdown product of AFB_1_ by culture supernatant of *P. aeruginosa* N17-1. Similar results were obtained by Alberts *et al.*, (2006) and Farzaneh *et al.*, (2012) [20,31]. It was suggested that AFB_1_ was possibly metabolized to degradation products with chemical properties different from that of AFB_1_ [20].

It indicated protein(s) involved in AFB_1_ degradation of *P. aeruginosa* N17-1 shared some common characteristics with those from *F. aurantiacum* and *S. maltophilia* 35-3, also they had some unique feature.

To conclude, a bacteria strain, *P. aeruginosa* N17-1, was isolated and characterized for its ability to efficiently degrade AFB_1_ and AFB_2_. An enzyme (enzymes) might be involved in the degradation process. Strain N17-1 shall be used as a new source of aflatoxin-degrading enzyme. The enzyme purification is underway in our lab.

## 4. Materials and Methods

### 4.1. Chemicals and Media

Aflatoxin B_1_ and B_2_ were purchased from Sigma-Aldrich. Standard solutions were diluted with methanol to make stock solutions at 50 ppm. Coumarin medium (CM) was prepared according to Guan *et al.* [22]. NB was used for liquid culture.

### 4.2. Bacteria Isolation

Two hundred and forty seven samples were collected from farm soils, maize and rice in June, 2013. Bacteria, which have the potential to degrade aflatoxin B_1_, were isolated using coumarin medium according to Guan *et al.* [22]. Colonies that were able to grow on coumarin medium were selected and further tested for AFB_1_ degradation.

### 4.3. Analysis of Aflatoxin Degradation

The selected isolates were cultured in NB for 12 h, then 1 mL culture broth was transferred to NB (20 mL) in a flask growing at 37 °C with agitation for 24 h in a shaker incubator. Then 0.1 mL AFB_1_ (500 ppb) solution was added to microbial cultures of 0.4 mL for final concentration of 100 ppb. The detoxification tests were conducted in the dark at 37 °C for 72 h. After incubation, cells were removed by centrifugation at 12,000 rpm for 5 min. Sterile NB was used to substitute microbial culture in the control.

Samples after treatment were extracted with chloroform according to the Association of Official Analytical Chemists [32]. The reaction mixtures were extracted three times with chloroform and the chloroform extracts were evaporated under nitrogen gas, the residue were dissolved in 50% methanol in water (1:1, *v*/*v*) and analyzed by HPLC. HPLC analysis was performed using a C18 (150 mm × 4.6 mm, 5 μm, Agilent). The mobile phase was methanol: water (1:1, *v*/*v*) at a flow rate of 1 mL/min. AFB_1_ was derived by a photochemical reactor (Waters, Milford, MA, USA) and measured by a fluorescence detector. The excitation and detection wavelengths were set at 350 and 450 nm, respectively.

The percentage of AFB_1_ degradation was calculated using the following formula:

(1 − AFB_1_ peak area in treatment/AFB_1_ peak area in control) × 100% (1)

Analysis of degradation of aflatoxin B_2_ and M_1_ was conducted by the same procedure as described above for AFB_1_.

### 4.4. Identification of Strain N17-1

General physiological and biochemical tests were carried out using previously described methods [33]. Genomic DNA of strain N17-1 was extracted using the method as described previously [34]. A universal primer set consisting of 27F and 1492R was used to amplify the 16S rRNA gene [35]. The nucleotide sequence was determined by direct sequencing and compared with available 16S rRNA gene sequence in the GenBank database using the BLAST program (National Library of Medicine, Bethesda, MD, USA). It was preserved at −80 °C before use.

### 4.5. Degradation of AFB1 by Cell Free Supernatant, Bacterial Cells and Intracellular Cell Extracts of Strain N17-1

The effects of cell free supernatant, bacterial cells and intracellular cell extracts of strain N17-1 on reduction of AFB_1_ were studied according to method as described previously [16,22,36]. Strain N17-1 was pre-cultivated in 4 mL NB at 37 °C for 12 h agitation at 180 rpm, and 1 mL of the culture was transferred to 100 mL of the same medium. After cultivation at 37 °C with shaking at 180 rpm for 48 h, the cells were harvested by centrifugation (5000× *g*, 10 min, 4 °C). The supernatant was collected and tested for AFB_1_ degradation.

The pellets were washed twice with phosphate buffer (50 mM, pH 7.0) before re-suspended in the phosphate buffer. The AFB_1_ degradation analysis was performed as described above. The phosphate buffer substituted bacterial cell suspensions in control.

Pellets were suspended in phosphate buffer (50 mM, pH 7.0; 3 mL buffer per gram cell mass). The suspension was disintegrated by using ultrasonic cell disintegrator (Ningbo Xinzhi Instruments Inc., Ningbo, China). The suspension was centrifuged at 12,000 *g* for 10 min at 4 °C. The supernatant was filtered aseptically using sterile filters of 0.22 μm pore size (Millipore, Darmstadt, Germany). The AFB_1_ degradation tests were performed as described above. Phosphate buffer substituted intracellular cell extracts in control.

### 4.6. Effects of Incubation Period, Temperature, Metal Ions and Proteinase K Treatment on Aflatoxins Degradation by Strain N17-1 Supernatant

0.1 mL AFB_1_ solution (500 ppb) was added to 0.4 mL culture supernatant in a 10 mL tube. For the optimal reaction or incubation time studies, the mixture was incubated in the dark at 37 °C without shaking for 1, 2, 12, 24, 48, 96, 120, 144 and 168 h, respectively. To determine the effect of temperature, the mixtures were incubated at 20, 30, 37, 45, 55 and 65 °C, respectively for 72 h. Ions, and protease effects were analyzed based on previous report of Guan *et al.* [22]. During the ions effects assay, 0.1 mL AFB_1_ (500 ppb) solution mixed with 0.4 mL culture supernatant for final concentration of 100 ppb was used as control. Super filter with cut-off molecular weight of 3 KD (Millipore, Darmstadt, Germany) was used for ultra filtration treatment on the culture supernatant.

### 4.7. Degradation Products Extraction and Detection

0.15 mL AFB_1_ (50 ppm) solution was added to microbial cultures of 1.35 mL for final concentration of 5 ppm. The detoxification tests were conducted in the dark at 37 °C for 72 h. The samples were extracted with chloroform, dried under nitrogen, suspended in methanol: water (7:3, *v*/*v*) and analyzed by LC-QTOF/MS. 1.35 mL NB media instead of culture supernatant of N17-1 was used as positive control, 0.15 mL methanol instead of AFB_1_ solution was set as negative control.

LC was performed on Agilent 1200 series HPLC (Agilent, Palo Alto, CA, USA) equipped with an auto injector and a quaternary HPLC pump. Chromatography was performed on a 2.1 × 150 mm inner diameter, 5 μm, Agilent Plus C18 column. A gradient separation was performed at 0.2 mL/min. Mobile phase A consisted of aqueous acetonitrile and mobile phase B consisted of 0.1% formic acid. The gradient profile was as follows, (a) 0→4 min, 40% A; (b) 4→10 min, 60% A; (c) 10→15 min, 60% A; (d) 15→20 min, 80% A; and (e) 20→40 min, 40% A. Total run time was 40 min. The sample injection volume was 20 μL. MS was performed with Agilent 6520 accurate-mass QTOF LC/MS (Agilent, Santa Clara, CA, USA). The optimized conditions were as follows: Compounds were analyzed in positive-ion mode. Capillary and fragmentor voltages were 3500 and 175 V, respectively, and the skimmer voltage was 65.0 V. The flow rate of drying gas was 10.0 L/min, and nebulizer was 40 psi. Nitrogen was used as the collision gas. Mass spectra were acquired in a full-scan analysis within the range of *m*/*z* 100−1000 using an extended dynamic range and a scan rate of 1.4 spectra/s and varying the collision energy with mass. The data station operating software used was the Mass Hunter Workstation software (version B.04.00, Agilent, Santa Clara, CA, USA). A reference mass solution containing reference ions 121.0508 and 922.0097 was used to maintain mass accuracy during the run time.

### 4.8. Statistical Analysis

Data were analyzed as a completely randomized single factor design by ANOVA with the general linear models procedure in SAS (SAS Institute, Cary, NC, USA). Significant *F* tests at the 0.05 levels of probability were reported. When a significant *F*-value was detected, Duncan’s Multiple Range Test was used to determine significant differences among means.

## 5. Conclusions

To conclude, a bacteria strain, *P. aeruginosa* N17-1, was isolated and characterized for its ability to efficiently degrade AFB_1_, AFB_2_ and AFM_1_. An enzyme (enzymes) might be involved in the degradation process. Strain N17-1 shall be used as a new source of aflatoxin-degrading enzyme. The enzyme purification is underway in our lab.

## Supplementary Materials

Supplementary materials can be accessed at: http://www.mdpi.com/2072-6651/6/10/3028/s1.

## Supplementary Files

Supplementary File 1
