# Correction: Sangare, L., *et al*. Aflatoxin B_1_ Degradation by a *Pseudomonas* Strain. *Toxins* 2014, *6*, 3028–3040

**DOI:** 10.3390/toxins7093538

**Published:** 2015-09-02

**Authors:** Lancine Sangare, Yueju Zhao, Yawa Minnie Elodie Folly, Jinghua Chang, Jinhan Li, Jonathan Nimal Selvaraj, Fuguo Xing, Lu Zhou, Yan Wang, Yang Liu

**Affiliations:** 1 Institute of Agro-products Processing Science and Technology, Chinese Academy of Agricultural Sciences, Beijing 100193, China; E-Mails: lancin.sangar@gmail.com (L.S.); zhaoyueju@caas.cn (Y.Z.); yelodieminnie@yahoo.fr (Y.M.E.F.); cjh2975@sina.com (J.C.); lijinhan1990@163.com (J.L.); sjonnim@gmail.com (J.N.S.); xingfuguo@caas.cn (F.X.); zhoulu@caas.cn (L.Z.); awangyan@126.com (Y.W.); 2 Key Laboratory of Agro-products Processing, Ministry of Agriculture, Beijing 100193, China

The authors are sorry to report that the legend of Figure 5 in their published paper [1] was incorrect. The correct figure legend should read:

**Figure 5.** LC-QTOF/MS profile of AFB_1_ degradation. (**A**) Electrospray ionization (ESI) total ion chromatogram (TIC) scan of positive control (0.15 mL AFB_1_ (50 ppm) solution was added to 1.35 mL NB medium for final concentration of 5 ppm. After incubation in the dark at 37°C for 72 h, the samples were extracted with chloroform); (**B**) ESI extracted ion chromatogram (EIC) scan of positive control; (**C**) ESI TIC scan of negative control (0.15 mL methanol was added to 1.35 mL culture supernatant of N17-1. After incubation in the dark at 37 °C for 72 h, the samples were extracted with chloroform); (**D**) ESI EIC scan of negative control; (**E**) ESI TIC scan of N17-1 treated sample (0.15 mL AFB_1_ (50 ppm) solution was added to 1.35 mL culture supernatant of N17-1 for final concentration of 5 ppm. After incubation in the dark at 37 °C for 72 h, the samples were extracted with chloroform); (**F**) ESI EIC scan of N17-1 treated sample.

We apologize for any inconvenience caused to the readers.
