# Supplementary material for: Aflatoxin B1 Degradation by a Pseudomonas Strain
Source: Toxins (Basel). 2014 Oct 23;6(10):3028–40. doi: 10.3390/toxins6103028 (PMC4210884; doi:10.3390/toxins6103028)
Supplement: Supplementary File 1 [file toxins-06-03028-s001.pdf]

Supplementary Information

Table S1. AFB_1 degradation by individual microbial isolates selected using coumarin medium.

No.	Isolate [a]	Source	Degradation (%) [b]
1	*Pseudomonas aeruginosa* N17-1	Soil from Changping, Beijing	82.8 ± 3.0
2	*Flavobacteriaceae* sp. 14	Soil from Quyang, Shanxi	74.1 ± 3.2
3	*Pseudomonas* sp. F26-1	Soil from Changping, Beijing	73.4 ± 4.0
4	19-A	Soil from Guangzhou, Guangdong	65.2 ± 4.4
5	NS10	Maize from Zhuozhou, Hebei	56.1 ± 4.5
6	NS6	Maize from Changzhi, Shanxi	52.7 ± 2.5
7	NS8	Maize from Baoji, Shannxi	45.6 ± 1.6
8	NS9	Maize from Yancheng, Jiangsu	41.4 ± 4.7
9	30	Soil from Shaoguan, Guangdong	39.4 ± 3.0
10	18	Soil from Fuyang, Anhui	30.4 ± 3.5
11	NSL 25	Rice from Huaian, Jiangsu	30.1 ± 1.7
12	16	Soil from Shantou, Guangdong	24.8 ± 2.8
13	29-D-1	Soil from Quyang, Shanxi	22.8 ± 1.9
14	20	Soil from Shaoguan, Guangdong	22.0 ± 1.6
15	NSL24	Rice from Huaian, Jiangsu	21.8 ± 2.2
16	M77	Maize from Suzhou, Anhui	21.5 ± 1.2
17	M30	Maize from Suzhou, Anhui	19.6 ± 3.6
18	25-C	Soil from Shaoguan, Guangdong	16.1 ± 0.1
19	G-B	Soil from Shantou, Guangdong	15.9 ± 0.3
20	NSL23	Rice from Yangzhou, Jiangsu	15.3 ± 2.5
21	25-B	Soil from Shaoguan, Guangdong	15.0 ± 0.1
22	M15	Maize from Suzhou, Anhui	13.9 ± 3.6
23	E-D	Soil from Guangzhou, Guangdong	12.8 ± 3.3
24	11-B	Soil from Dianbai, Guangdong	11.7 ± 0.1
25	F4-1	Soil from Changping, Beijing	11.0 ± 1.3

[a] Individual microbial isolates grown on medium with coumarin as the sole carbon source; [b] The detoxification tests were conducted in the dark at 37 °C for 72 h. The percentage of AFB_1 degradation was calculated using the following formula: (1 − AFB_1 peak area in treatment/AFB_1 peak area in control) × 100%.

Table S2. Characteristics of strain N17-1.

Item	Result [a]	Item	Result	Item	Result
Cell shape	rod-shaped	D-Salicin	−	D-Glucuronic	+
Gram stain	−	*N*-Acetyl-D-Glucosamine	+	Glucuronic acid amide	+
Catalase activity	+	*N*-Acetyl-β-D-Mannosidase	−	Mucic	−
Oxidase activity	+	*N*-Acetyl-D-Galactosamine	−	Quinine acid	+
Spore formation	−	*N*-Acetyl-Ceramide	−	*p*-Hydroxy acid	+
Nitrate reduction	+	α-D-Glucose	+	Methyl pyruvate	+
Hydrolysis of casein	+	D-Mannose	−	D-Methyl lactate	−
Hydrolysis of starch	+	D-Fructose	−	L-Lactate	+
**API20E**		D-Galactose	−	Citrate	+
Utilization of citrate	+	D-Serine	−	α-Ketoglutarate	+
Production of Indole	−	D-Fucose	+	Biolog GEN III (Chemical sensitivity tests)	
Production of H_2S	−	Sugar	−	Positive control	+
Voges-Proskauer test	−	L-Rhamnose	−	Growth at pH 6.0	+
Arginine double hydrolase	+	Inositol	−	Growth at pH 5.0	+
Tryptophan deaminase	−	Glycerin	+	Growth at 4% NaCl	+
Urease	+	D-Glucose-6-phosphate	+	Minocycline	+
Lysine decarboxylase	−	D-Fructose-6-phosphate		1% Lactate	+
**Biolog GEN III (Growth experiments)**		D-Aspartic acid		Fusidic acid	+
Negative control	−	Gelatin	+	D-Serine	+
L-Galacturonic acid lactone	−	Stachyose	−	Troleandomycin	+
D-Maltose	−	L-Alanine	−	Rifamycin SV	+
D-Trehalose	−	L-Arginine	+	Lincomycin	+
D-Cellobiose	−	L-Aspartate	+	Guanidine hydrochloride	+
Gentiobiosyl	−	L-Glutamate	+	Tetradecyl sulfate	+
β-hydroxy-D, L-butyric acid	+	L-Histidine	+	Vancomycin	+
Glycine-L-proline	+	L-Pyroglutamate	+	Tetrazolium violet	+
3-methyl-D-glucose	−	Turanose	−	Nalidixic acid-resistant	+
D-Raffinose	−	Pectin	−	Potassium tellurite	+
α-D-Lactose	−	Dextrin	−	Aztreonam	+
D-Melibiose	−	Sugar acid	−	Sodium butyrate	+
β-Methyl-D-Glucamine	−	D-Gluconate	+	Blue tetrazolium	+

[a] "+" means positive response; "–" means negative response.
